# Structural Pathways of Cytokines May Illuminate Their Roles in Regulation of Cancer Development and Immunotherapy

**DOI:** 10.3390/cancers6020663

**Published:** 2014-03-25

**Authors:** Emine Guven-Maiorov, Saliha Ece Acuner-Ozbabacan, Ozlem Keskin, Attila Gursoy, Ruth Nussinov

**Affiliations:** 1 Center for Computational Biology and Bioinformatics and College of Engineering, Koc University, Rumelifeneri Yolu, 34450 Sariyer Istanbul, Turkey; E-Mails: eguven@ku.edu.tr (E.G.-M.); sozbabacan@ku.edu.tr (S.E.A.-O.); okeskin@ku.edu.tr (O.K.); agursoy@ku.edu.tr (A.G.); 2 Cancer and Inflammation Program, Leidos Biomedical Research, Inc., Frederick National Laboratory for Cancer Research, National Cancer Institute, Frederick, MD 21702, USA; 3 Sackler Institute of Molecular Medicine, Department of Human Genetics and Molecular Medicine, Sackler School of Medicine, Tel Aviv University, Tel Aviv 69978, Israel

**Keywords:** structural pathways, cytokines, structures of cytokines, structural pathways of cytokines, protein interactions, protein interaction prediction, cancer immunotherapy

## Abstract

Cytokines are messengers between tissues and the immune system. They play essential roles in cancer initiation, promotion, metastasis, and immunotherapy. Structural pathways of cytokine signaling which contain their interactions can help understand their action in the tumor microenvironment. Here, our aim is to provide an overview of the role of cytokines in tumor development from a structural perspective. Atomic details of protein-protein interactions can help in understanding how an upstream signal is transduced; how higher-order oligomerization modes of proteins can influence their function; how mutations, inhibitors or antagonists can change cellular consequences; why the same protein can lead to distinct outcomes, and which alternative parallel pathways can take over. They also help to design drugs/inhibitors against proteins *de novo* or by mimicking natural antagonists as in the case of interferon-γ. Since the structural database (PDB) is limited, structural pathways are largely built from a series of predicted binary protein-protein interactions. Below, to illustrate how protein-protein interactions can help illuminate roles played by cytokines, we model some cytokine interaction complexes exploiting a powerful algorithm (PRotein Interactions by Structural Matching—PRISM).

## 1. Introduction

**Figure 1:**
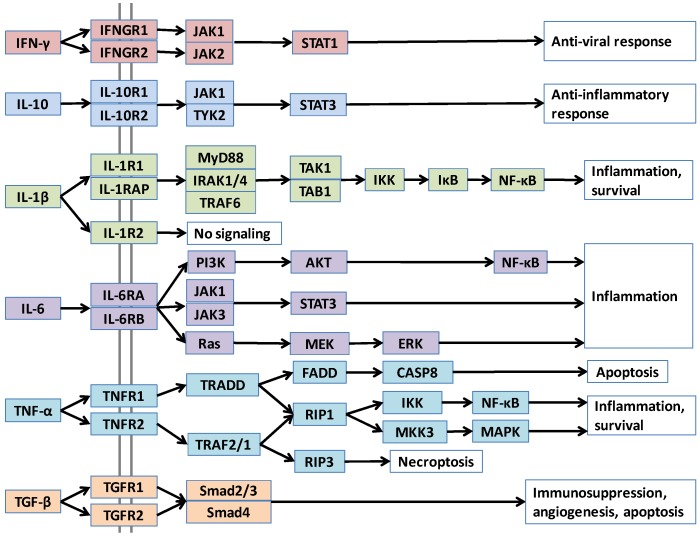
Pathways of major cytokines, in node-and-edge representation.

Interactions of proteins with themselves, with other proteins, and other small and large macromolecules define their functions. Abstract diagrams of protein interactions—where nodes are proteins and edges are interactions—are useful: they provide the cellular “master plan”; that is, a broad view of cellular signaling pathways and their circuitry [1,2]. Nonetheless, these simple representations that depict which proteins interact, lack the structural detail which allows understanding of how proteins interact, how large signalosome complexes assemble, under which conditions distinct parallel pathways are activated, and how oncogenic mutations in a protein influence its interactions, its pathways and ultimately, cellular behavior [3,4,5,6,7,8,9,10]. Interaction details are also critical for drug discovery [11]. Combined with biochemical and biological data, structural pathways can help predict global drug effects at the cell, tissue and organism levels [12,13]. Structural pathways can predict new interactions that have not yet been shown experimentally; these new interactions may shed light on why aberrations in a given protein may result in distinct outcomes. Atomic details of protein complexes assist in understanding cellular control which can modify signaling processes. As a case in point, both interleukin-10 (IL-10) and IL-6 activate STAT3 (Signal transducer and activator of transcription 3) transcription factor; however, the former leads to anti-inflammatory response while the latter initiates inflammation (Figure 1). Structural predictions can identify and help elucidate the functional mechanisms. Here, we map cancer-related structural pathways of cytokines, and focus on examples illustrating the usefulness of structural data in understanding cytokine function; the role of aberrant cytokine expression in pathogenesis of autoimmune diseases and cancer; the importance of oligomerization modes of cytokines and their receptors; and the benefit of viral cytokine antagonists in designing peptide inhibitors of cytokines. Cytokines have critical roles in tumor development; construction of their structural pathways can help us understand the mechanism of their action in a tumor microenvironment. 

Cytokines are complex messengers that enable crosstalk between tissues and the immune system. They also underlie events in tumor initiation and progression [14]. Cytokines, their receptors, downstream consequences, as well as their upstream pathways that promote cytokine production are important in malignant transformation. Figure 1 displays node and edge networks of some major cytokines. Structural details of these pathways can provide novel insights to the mechanisms of cytokine-induced oncogenesis. 

Cytokines are grouped into nine categories: chemokines, interferons (IFNs), tumor necrosis factors (TNFs), transforming growth factor-β (TGF-β) family members, interleukin-1 (IL-1) family members (such as IL-1α, IL-1β, and IL-18), IL-10 family members, IL-17 family members, hematopoietic growth factors (such as IL-1, G-CSF, GM-CSF), and platelet derived growth factors (PDGFs) [15]. Cytokine receptors are classified into seven subclasses: Type-I cytokine receptors (IFN-α, IFN-β, G-CSF, GM-CSF, *etc*.), Type-II cytokine receptors (IFN-GR IL-10R), TGF-β receptors, TNF receptors, immunoglobulin superfamily receptors (IL-1R), G-protein coupled receptors, and IL-17 receptors [16].

Cytokines execute their function by binding to their receptors on target cells and activating downstream pathways. Pathways merge and diverge at several points with more than one parallel downstream path, as in the case of TNF-α, IL-1β and IL-6 in Figure 1. A cytokine can also bind to different receptors with varying affinities. Differences in receptors’ concentration, orthosteric or allosteric mutations that alter the affinity of the cytokine to the receptors, all can lead to distinct consequences. Structural details can capture differences in affinity and efficacy. Further, not only alterations in downstream pathways, but also variations in cytokine concentrations due to upstream changes can impact cellular outcomes. Activation of oncogenes, such as Ras, results in inflammatory cytokine expression [17,18]. Constitutive activation of NF-κB, the major pathway that regulates immune inflammatory responses [19] and produces pro-inflammatory cytokines, such as TNF-α, IL-1β, and IL-6 [15,20], leads to chronic inflammation, which promotes cancer [1,21,22]. Inhibition of NF-κB in tumor cells switches inflammation-induced tumor growth to regression and has been suggested as an effective therapy [19]. Lipopolysaccharide (LPS) of Gram-negative bacteria activates the Toll-like receptor (TLR) pathway, which stimulates the NF-κB family of transcription factors [1]. LPS causes metastatic tumor growth of colon adenocarcinoma in the lung in a mouse model and NF-κB is responsible for this action [19]. Thus, upstream pathways control the cytokine concentrations in cells and have vital roles in cancer development. Our aim in this review is to provide an overview of cytokines in cancer from a structural standpoint. Below, we continue with a broad description of cytokines and their roles in cancer development. Then, we briefly introduce computational strategies to model protein-protein interactions and describe the PRISM tool that we use to construct structural pathways. We follow by providing structural case studies of major cytokines, showing the usefulness of structures in characterizing cytokine function and regulation, and understanding mechanisms of mutations and inhibitors/antagonists in cytokine signaling. While there are additional cytokines which are important in tumor development, here we focused on widely studied ones.

## 2. Cytokines and Their Roles in Cancer

Cytokines are a diverse group of signaling molecules that are produced as a result of infection, inflammation, injury, and cellular stress [23]. They activate immune cells and regulate their development, differentiation, and migration; they stimulate host immune responses to control stress and minimize cellular damage, and thus restore homeostasis [15,19,23]. These messenger proteins are either secreted or are membrane-bound, allowing communication of immune cells with each other in a paracrine, autocrine or endocrine fashion [15,16]. Membrane-bound forms of cytokines allow communication through direct cell-cell contact, but secreted cytokines permit rapid spread of the signal to other tissues [16]. They act locally or systemically to coordinate acute or chronic inflammatory responses.

Cytokines have complex and even opposing roles in the immune system [16]. They are either pro- or anti-inflammatory. Pro-inflammatory cytokines, such as IL-1β and TNF-α, induce inflammation as a result of infection or injury; anti-inflammatory cytokines, like IL-10 and TGF-β, suppress the activity and production of pro-inflammatory signals [20], limiting inflammation and host damage. While not the individual cytokines, their overall balance determines cell fate: stimulate inflammatory response or suppress it [20]. Different cytokine combinations give rise to distinct consequences, such as inflammation, proliferation, and angiogenesis [15]. Imbalances in cytokine expression or signaling contribute to malignant transformation [23]. 

Cytokines stimulate host immune responses not only against pathogens, but against tumors [16]. Host-derived cytokines can inhibit tumor progression as well as promote proliferation, decrease apoptosis, and foster invasion and metastasis [23]. They are important orchestrators of cancer-related inflammation [14,17]. Inflammation is required to combat pathogens, heal wounds and maintain tissue homeostasis [21,24]; if not finely tuned, it can lead to oncogenesis [1]. Inflammation has roles in almost all phases of tumor development, including tumor initiation, promotion and metastasis [21,25,26]. Tumor cells perturb tissue cytokine expression and these alterations in cytokine levels recruit infiltrating leukocytes to the tumor microenvironment, which in turn contribute to extra cytokine production [18,21,23,27]. Cytokines are pleiotropic, allowing a particular cytokine to act on various cell types; they may also have opposite effects on distinct target cells [15,20,23], depending on other cytokines present in the local environment. Cytokines are redundant, with multiple cytokines having similar activities [15,20,23,28], allowing them to compensate for each other in deficiency or drug resistance.

The immune system has dual roles in cancer: it eradicates cancer cells when it recognizes tumor antigens and provokes anti-tumor immune response. On the other hand, the immune system selects for resistant cancer cells best fit to survive in immunocompetent hosts and promote its growth, which is also called “cancer immunoediting” [24]. During primary tumor development, immune surveillance eliminates tumors, but established cancers provoke immune tolerance [29,30]. Tumors in immunodeficient host are “unedited” and more immunogenic; when transplanted to another host with intact immune system, they alert anti-tumor immunity. However, they are “edited” in immunocompetent host, allowing them to escape the immune system [24,29,30].

Cytokines play critical roles in the pathogenesis of autoimmune and auto-inflammatory diseases and cancer. Nevertheless, they are also used as therapeutic agents in such diseases. Much effort has been invested in cancer immunotherapy, exploiting cytokines in cancer treatments to activate immune responses against cancer [16]. Manipulation of cytokine levels can be used to stimulate immune responses [23]. Several cytokines have been used in clinical trials, and Type-I interferons (IFNs) were proposed to be beneficial for treatment of cancer [16]; however, cytokine redundancy hinders such efforts [16].

In order to investigate the role of cytokines in carcinogenesis, cytokine-deficient mice were used in several studies [23,30,31,32,33,34,35]. For instance, lack of IL-1 decreases tumor invasiveness and angiogenesis [16] and IL-1 receptor (IL-1R) antagonists suppress tumor growth [35]. TNF-α-deficient mice are resistant to skin tumor [33]. Thus, pro-inflammatory cytokines IL-1 and TNF-α promote some tumor types. In contrast, IFN-γ contributes to prevention of carcinogen-induced sarcomas [30] and its loss enhances tumor formation [23]. IL-10 deficiency increases IL-1 levels, promoting cancer in the absence of IL-10 [36]. Thus, IFN-γ and anti-inflammatory cytokine IL-10 protect the host against cancer. However, the inter-relationship between pro- and anti-inflammatory cytokines and oncogenesis is still not entirely clear. TGF-β acts to inhibit cancer in normal and pre-cancerous cells, although cancer cells lose tumor-inhibitory effects of TGF-β, invade and metastasize to other tissues [37]. 

## 3. Approaches in Construction of Structural Pathways

Structural information helps to understand cellular signaling pathways in detail. However, in many cases, structural knowledge relating to protein-protein interactions is missing, as in the case of TNF-α-TNFR1. In order to address this problem, several computational structural approaches have been developed to model protein interactions. Strategies include *ab initio* docking and template-based techniques. Docking approaches may or may not exploit prior knowledge of protein interactions [38]. Most of the traditional docking techniques treat proteins as rigid bodies with minimal flexibility [3] and aim to dock proteins with complementary surfaces and electrochemical properties. They generate many false-positives because complementary surfaces are often found between target proteins. In addition, they take more CPU time, which can make them impractical for proteome-scale studies. In contrast, template based techniques make use of prior protein-protein interaction knowledge. Binding surfaces or interfaces of proteins are more conserved among different unrelated protein folds the rest of protein surfaces [39,40]. That is, although the global structures of proteins may differ, they may use similar interfaces to interact with their partners. Template-based techniques are more suitable for large-scale studies in terms of CPU time. A powerful template-based algorithm developed by our group, PRotein Interactions by Structural Matching (PRISM) employs recurring interfaces of protein-protein interactions whose 3D structures were previously resolved and are available in the PDB as knowledge-based template motifs [41,42]. In the cytokine case studies described below, we used the PRISM algorithm to model missing structures of protein-protein complexes, such as TNF-α-TNFR1 and IL10-IL10RB complexes. Computational tools help enrich the available structural data for protein interactions and analyze the effects of mutations on the interactions and pathways.

## 4. Case Studies

### 4.1. IL-1β

**Figure 2:**
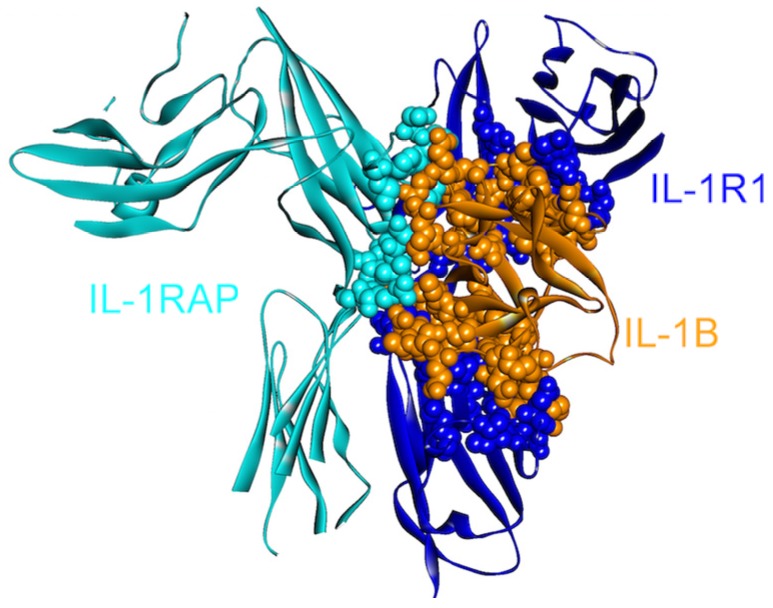
The structure of IL-1β, IL-1R1 and IL-1RAP complex (PDB Code_Chains: 4dep_DEF). These are all-beta proteins. There are three interfaces in the complex: one between IL-1RAP/IL-1R1, and the others between IL-1B/IL-1R1 and IL-1B/IL-1RAP. Atoms of interacting residues are represented as balls in order to highlight the interface regions.

**Figure 3:**
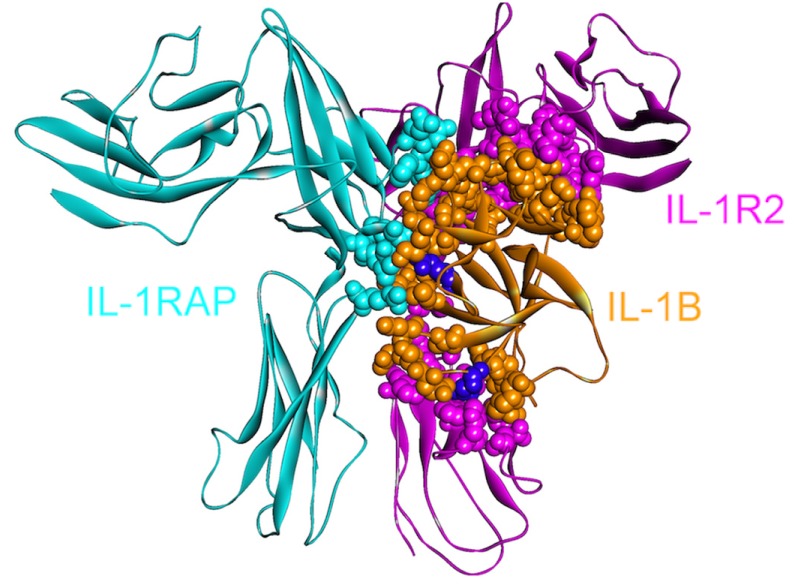
The structure of IL-1β, IL-1R2 and IL-1RAP complex (PDB Code_Chains: 3o4o_ABC). Atoms of interface residues are represented with balls. There are many common residues on IL-1β that binds to IL-1R1 and IL-1R2. Purple residues (Gln48 and Glu111) are computational hot spots and are specific to IL-1R2 and IL1RAP binding, respectively.

IL-1β can activate its downstream target cells by forming a signaling complex with two membrane-bound receptors: IL-1 receptor type I (IL1R1) and IL-1 receptor accessory protein (IL-1RAP/IL1RAcP). Since it is crucial in mediating the inflammatory response, IL-1β signaling is strictly regulated through two receptors: a “decoy” receptor IL-1RII (IL-1 receptor type II, IL1R2) and a receptor antagonist IL-1RA [43]. Although IL1R2 does not have an intracellular TIR domain, which is necessary for signaling [44], the extracellular regions of IL1R1 and IL1R2 are homologous, permitting efficient binding of the IL-1β ligand to both receptors [43] essentially through the same residues (Figure 2 and Figure 3). IL-1β signaling can be blocked by the decoy receptor IL1R2 either by preventing the interaction of IL-1β with IL1R1 through competitive binding [45,46] or by decreasing the concentration of IL1RAP, which is an essential member of the signaling complex, by forming a non-signaling complex with IL-1β and IL1R2 [47,48] (Figure 2 and Figure 3).

IL1R2, the decoy receptor, is upregulated in pancreatic and ovarian cancer [49,50]. The immune system induces apoptosis; however, this decoy receptor can protect pancreatic cancers [51] by blocking IL-1 signaling. Targeting IL1R2 is considered effective for inhibiting tumor angiogenesis [50] as IL-1 is essential in tumor angiogenesis and invasiveness [34]. While inhibiting the IL-1β and IL1R2 interaction is a therapeutic aim, it is challenging since the extracellular domains of IL1R1 and IL1R2 are homologous and IL-1β uses essentially the same residues for binding. When the structures of the signaling and non-signaling complexes of IL-1β (PDB Codes: 4dep and 3o4o, respectively) are compared, a few—though crucial—differences can be observed. Computational hot spot residues on IL-1β specific to IL1R2 and IL1RAP binding include Gln48 and Glu111, respectively, which may be specifically targeted by drugs with the aim of inhibiting these interactions (Figure 3). Other residues specific to IL-1β—IL1R2 binding consist of Ser13, Ser21, Tyr24, Lys27, Asp35 and Asn129; whereas Lys109 is specific for IL-1β—IL1RAP binding.

### 4.2. TNF-α

TNF-α, a pro-inflammatory cytokine plays a fundamental role in inflammation and host defense [52,53,54]. TNF-α is found both as soluble and membrane-bound forms. Its overexpression is seen in several chronic inflammatory diseases such as Crohn’s disease and ulcerative colitis and also in cancer [15,53,55]. Therefore, there are many studies on treatment of such diseases with anti-TNF therapies, including anti-TNF antibodies, soluble TNF receptors (TNFRs), and small molecule inhibitors [54,55,56].

TNF-α signals through two receptors: TNFR1 (also known as p55 or TNFRSF1A) and TNFR2 (also known as p75 or TNFRSF1B), which stimulate two distinct intracellular pathways as can be seen in Figure 1. TNFR1 is ubiquitously expressed in almost all cells, but TNFR2 expression is restricted to certain cell types, such as T-lymphocytes, endothelial cells, oligodendrocytes and some neuron subtypes [53]. The extracellular parts of these two receptors are similar but their cytoplasmic parts are different. TNFR1 possesses a death domain that allows interaction with other death domain containing proteins like TNFR1-associated death domain (TRADD) and Fas-associated death domain (FADD), whereas TNFR2 does not [53]. This variance in their cytoplasmic parts leads to interaction with different proteins and distinct interaction partners will result in diverse cellular outcomes. 

TNF-α mostly signals through TNFR1, possibly because it is ubiquitously expressed. TNFR1 activation induces apoptosis via TRADD and FADD and inflammation. On the other hand, TNFR2 recruits TRAF2 and TRAF1 (TNF-receptor associated factors) and activates NF-κB and expression of pro-survival genes [53]. Since TNFR2 does not have a death domain, it cannot interact with death domain containing adaptors. TNFR1 and TNFR2 downstream pathways also crosstalk (Figure 1) and they have certain overlapping functions.

**Figure 4:**
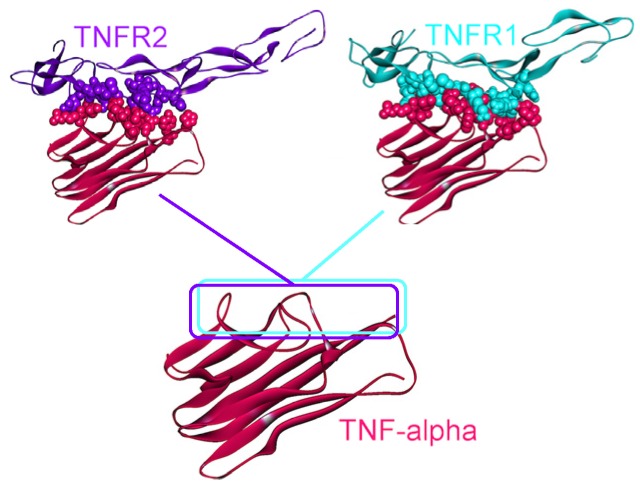
TNFR1 and TNFR2 bind to the same site on TNF-α, thus they are mutually exclusive. The structure of the TNF-α and TNFR2 complex is available in the PDB (PDB Code_Chains: 1alq_AR), however, we modeled the TNF-α and TNFR1 interaction by PRISM [41]. Atoms of interface residues are represented with balls. Solid lines and rectangles indicate the position of interfaces on TNF-α. Since the rectangles overlap almost completely, these two interactions cannot co-exist.

The 3D structure of TNF-α-TNFR2 complex is available in the PDB (PDB Code_Chains: 1alq_AR) [57], but the TNF-α-TNFR1 structure has not yet been solved. We modeled this interaction by PRISM [41], using the template interface 1tnrAR, which is the interface between TNF-β-TNFR1 [58]. As shown in Figure 4, interfaces of the interactions of TNF-α with its receptors almost fully overlap. Therefore, these two interactions are competitive; either TRADD is recruited to the receptor, or TRAF2.

**Figure 5:**
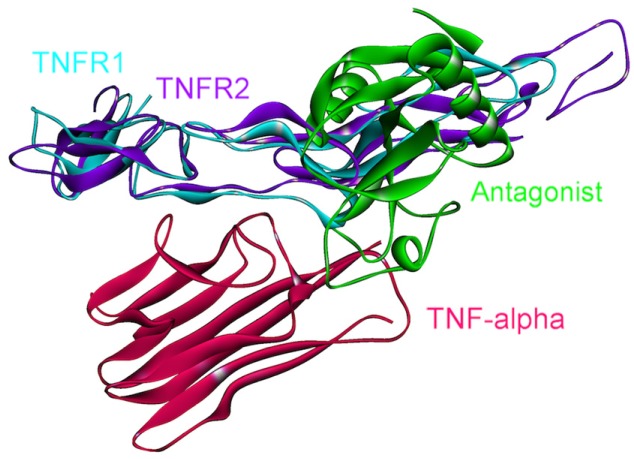
Receptors and antagonist bind to the same site on TNF-α. Therefore, if an antagonist occupies the interface on TNF-α, TNFRs can no longer bind there and thus TNF signaling is blocked. The complex structure of TNF-α and antagonist is available in the PDB (PDB Code_Chains: 3l9j_CT).

**Figure 6:**
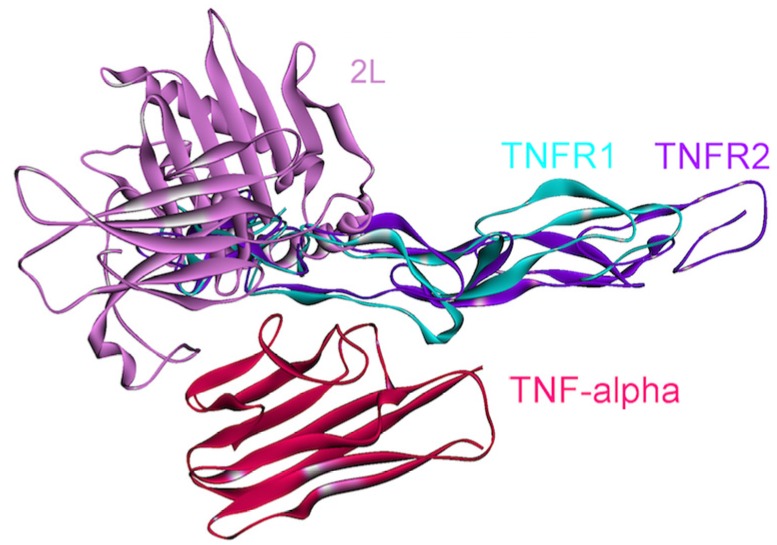
Receptors and the poxvirus 2L protein bind to the same site on TNF-α. Therefore, the 2L protein inhibits the association of TNF-α with its receptors, and thus its signaling. The complex structure of TNF-α and 2L is available in the PDB (PDB Code_Chains: 3it8_AD).

Some TNF-α mutants/variants (PDB Codes: 2e7a and 2zpx), in which six receptor-binding residues are substituted, specifically bind to TNFR1, but not to TNFR2 and inhibit its signal transduction [52,54]. We note that even though the authors did not observe interactions between these mutant TNF-α’s and TNFR2, PRISM suggested this interaction, with even more favorable energy than the interaction with TNFR1. This difference may stem from the fact that crystal structures typically do not reflect the physiological conditions. There are ample data that environmental conditions shift the protein populations. Data indicate that when crystallized, forms differing from those prevailing *in vitro* or *in vivo* may be captured [59]. This may explain why PRISM still finds this interaction with the mutant while the interaction did not take place *in vitro*. In addition, some antagonist proteins 2L (poxviral protein) [60] and CTLD (C-type lectin-like domain) [55] (PDB Codes: 3it8 and 3l9j, respectively), also bind to TNF-α at the interface between TNF-α and the receptors, thus interfering with its receptor-binding and signaling (see Figure 5 and Figure 6). Thus, structural details of interactions can provide insight into the mechanisms of mutant proteins or antagonists blocking or altering downstream pathways. Also, these structures of the complexes with the antagonists can assist pharmaceutical efforts to develop anti-TNF therapeutics for treatment of autoimmune and chronic inflammatory diseases, such as Crohn’s disease. 

**Figure 7:**
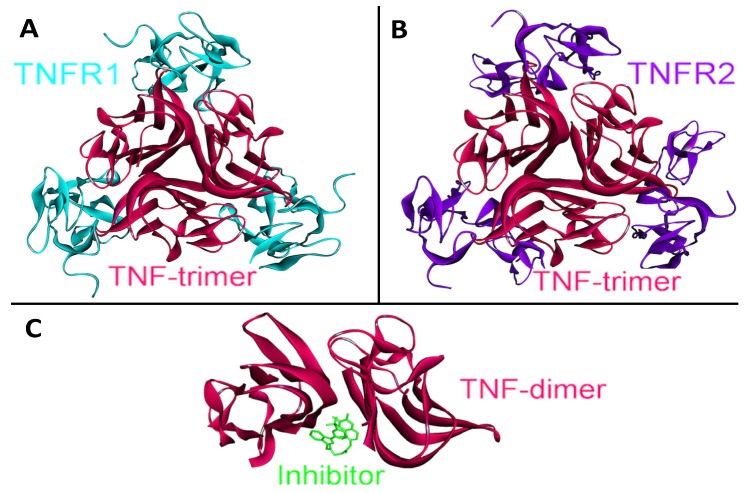
The structure of TNF-α homotrimer and homodimer. TNF-α should form homo-trimers in order to execute its function. TNF-α homotrimer has 3-fold symmetry (PDB Code_Chains: 1alq_ABC). (**A**) Trimeric TNF-α and TNFR2 complex is available in PDB (PDB Code_Chains: 1alq_ABCRST); (**B**) We obtained trimeric TNF-α and TNFR1 complex by superimposition of monomeric interaction predicted by PRISM [41] with the available trimeric structure (PDB Code_Chains: 1alq_ABC); (**C**) A small molecule inhibitor binds to TNF-α and prevents homo-trimerization, although it allows dimerization (PDB Code_Chains: 2az5_ABA).

Function and signaling of TNF-α does not depend on only its interactions with the receptors; its higher order oligomerization modes also play essential roles in TNF-α signaling. TNF-α performs its functions by forming homo-trimers with a 3-fold symmetry (PDB Code_Chains: 1alq_ABC), each TNF-α monomer interacting with a TNFR1 or TNFR2 (see Figure 7, parts A and B) [57]. 

We obtained trimeric TNF-α-TNFR1 by superimposition of monomeric interaction predicted by PRISM [41] and the available structure of the trimeric TNF-α-TNFR2 complex (PDB Code_Chains: 1alq_ABC). Moreover, panel C in Figure 7 displays a small molecule inhibitor binding to TNF-α and preventing its homo-trimerization, although this inhibitor still allows dimerization (PDB Code_Chains: 2az5_ABA). This molecule not only blocks TNF-α trimerization; it also blocks its signaling [56]. Thus, higher order oligomerization of TNF-α may be as important as its interactions with receptors in TNF-α signaling. Structural pathways can capture the oligomerization modes of proteins and their altered interactions due to a mutation, inhibitor or antagonist binding, which is not the case in pathway diagrams. 

### 4.3. IL-6

**Figure 8:**
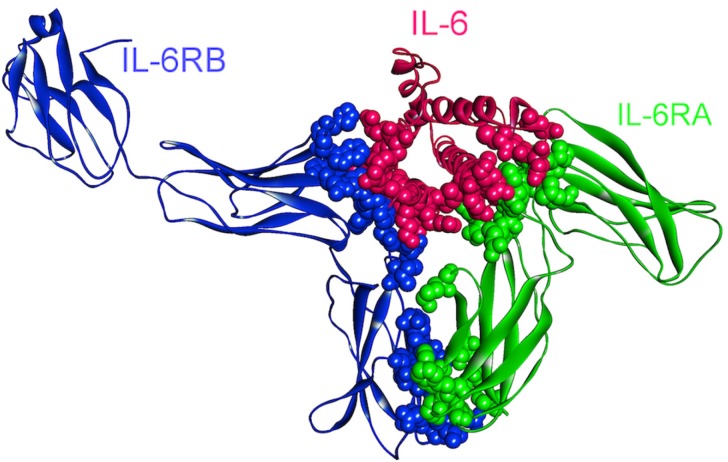
The structure of IL-6, IL-6RA, and IL-6RB complex (PDB Code_Chains: 1p9m_ABC). Atoms of interface residues are represented with balls.

Intracellular IL-6 signaling initiates through the association of IL-6–IL-6R (IL6RA, CD126) complex with gp130 (IL6RB, CD130) protein (Figure 8) and induction of gp130 dimerization [61] resulting in a hexameric complex capable of signaling [62]. A soluble form of IL-6R (sIL-6R) was also found in body fluids [63,64] and its affinity to bind to IL-6 (similar to membrane bound IL-6R) leads to the IL-6 trans-signaling process so that the IL-6–sIL-6R complex can stimulate gp130 expressing cells, although they lack membrane bound IL-6R [65,66,67]. Experiments showed that IL-6 signaling can act both as pro- and anti-inflammatory/regenerative depending on whether IL-6 trans-signaling or classical signaling takes place, respectively [68,69,70,71,72]. 

Initiated IL-6 signaling can activate JAK/STAT [61], Ras-Raf [73] and PI3K/AKT signaling pathways (Figure 1) [74,75]. IL-6 has anti-apoptotic and pro-tumorigenic activities regulated by these different pathways [76]. Due to being one of the most ubiquitously deregulated cytokines in cancer, IL-6 is shown to modulate growth and differentiation in several malignant tumor cells [77,78] and its overexpression with its receptors (IL-6R and sIL-6R) has been found in a wide range of cancers [76]. IL-6–JAK–STAT signaling path has been found to be important for tumorigenesis in various tumor models such as ovarian, lung, bladder, breast and colon cancers [76]. 

Targeting IL-6 signaling pathway in cancer therapy is supported by a numerous preclinical and translational observations. The pathological role of IL-6 in cancer has also been validated with successful treatment of certain diseases with drugs inhibiting IL-6 signaling [76]. Currently, several monoclonal antibodies (mAbs) and mAb mixtures against IL-6 and IL-6R (in preclinical models) are being developed with encouraging results in cancer cell lines and animal models [79,80,81,82]. Additionally, small molecule compounds for the inhibition of IL-6 or downstream proteins are also developed and being evaluated in preclinical and clinical cancer models [74].

There are 14 oncogenic mutations (from the COSMIC database [28,83]) that correspond to residues on the binding interfaces of IL-6–IL-6R–gp130 hexameric complex (Figure 8) and these mutations are related mostly to colon cancer (7 mutations), and also endometrial (2), liver (2), lung (1), breast (1) and stomach (1) cancers. Based on the fact that IL-6 and its receptors are overexpressed in various cancer types, these mutations are speculated not to be loss-of-function mutations, with the complex being turned on constitutively. 

### 4.4. IL-10

IL-10 is an important immunomodulatory cytokine that suppresses expression of pro-inflammatory cytokines, such as IL-1, IL-6, and TNF-α, by inhibiting NF-κB transcription factors [24]. IL-10 also blocks the development of regulatory T cells (T_reg_s) and Myeloid-derived suppressor cells (MDSCs), which are inhibitors of anti-tumor immunity [14,17,84]. In the presence of IL-10, tumor-specific immunity can be activated, but suppression of IL-10 signaling contributes to tumor progression by inhibiting anti-tumor immunity and allowing excessive inflammation that promotes cancer. IL-10 has two receptors—IL-10RA and IL-10RB—both of which are required for IL-10 signaling [85,86].

**Figure 9:**
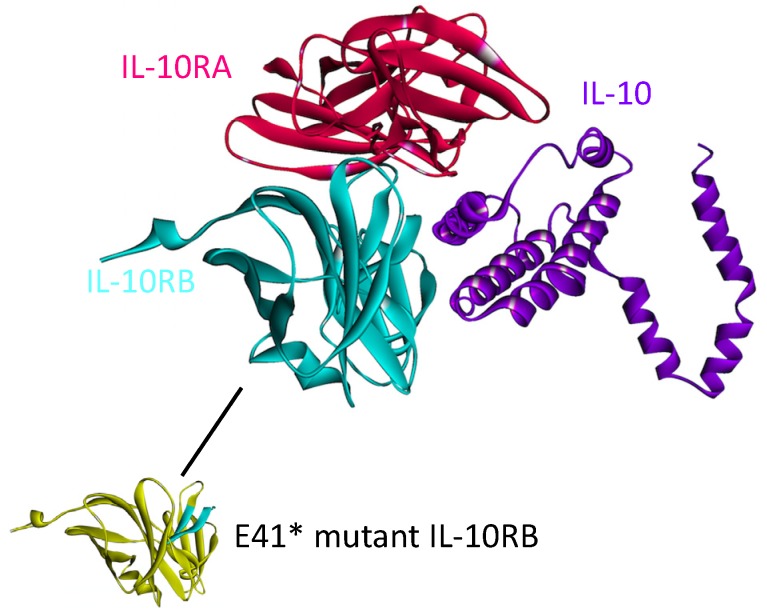
The structure of the IL-10, IL-10RA and IL-10RB complex, obtained by superimposition of IL10-IL10RA and IL10-IL10RB complexes. The structure of the IL10-IL10RA is available in the PDB (PDB Code_Chains: 1y6k_LR) but interaction of IL-10 (PDB Code_Chain: 2ilk_A) with IL-10RB (PDB Code_Chain: 3lqm_A) was modeled by PRISM [41] in our previous study [87]. IL-10 needs to bind to both IL-10RA and IL-10RB simultaneously to propagate the signal downstream. The yellow region on IL-10RB below, would be gone in E41* mutant form of IL-10RB and this mutation abolishes the interaction of IL10-IL10RB since the majority of the interface would be missing.

The structure of the complex between IL-10 with IL-10RA is available in the PDB (PDB_ID: 1y6k_LR). However, the IL10-IL10RB interaction has not yet been solved. We modeled this interaction by PRISM [41] in our recent work on the structural IL-10 network [87]. IL-10 needs to dimerize and bind to both of its receptors simultaneously in order to transduce the signal [88]. According to our model, IL-10 can interact with both receptors at the same time, without a steric clash (see Figure 9). 

This complex structure elucidates the mechanisms of some cancer mutations, such as E41* nonsense mutation on IL-10RB, which is a clinically observed mutation in adenocarcinoma with 2% frequency [89]. This mutation abolishes IL10-IL10RB interaction [87] and thus prevents IL-10 signaling. Prevention of IL-10 signaling allows excessive inflammation and promotes development of T_reg_s and MDSCs, which inhibit anti-tumor immunity and permit tumor growth [84]. 

### 4.5. TGF-β

The anti-inflammatory cytokine TGF-β superfamily regulates key processes such as development, and its deregulation is involved in the pathogenesis of various diseases including cancer [90]. Being a growth inhibitor, TGF-β functions in normal tissue homeostasis. It has three isoforms (TGF-β1, TGF-β2 and TGF-β3) [91]. Active ligands TGF-β1 and 3 bind to the transmembrane TGF-β type II receptor (TβRII, TGFBR2) with high affinity and selectivity, subsequently activating and recruiting the TGF-β type I receptor (TβRI, TGFBR1, ALK5) into the complex [92]. Smad signaling, the main downstream signaling pathway is then initiated via the phosphorylation of receptor-associated Smads (Smad 2 and 3) by the activated TβRI. The activated Smad 2/3 and Smad 4 then form a stable complex (Figure 1), which translocates into the nucleus [92].

Under pathological conditions such as cancer progression, intracellular signaling networks of tumor cells are disrupted by tumor suppressor loss and oncogenic mutations through inhibition of the TGF-β homeostatic function [92]. TGF-β signaling has been shown to have two opposing roles in cancer: tumor suppression and tumor promotion. The tumor suppressing effect of TGF-β signaling is observed in the early stages of tumorigenesis, whereas tumor progressing effects are emphasized once the tumor is developed, when carcinoma cells gain oncogenic mutations in order to resist the growth inhibition mediated by TGF-β; under these circumstances TGF-β expression increases in the tumors [92,93]. TGFBR1, TGFBR2, SMAD4 and SMAD2 are the most commonly mutated genes in TGF-β signaling pathway particularly in colon, pancreas and gastric cancers [93]. The mutations are loss of function, implying that the disruption of TGF-β signaling has roles in those types of cancers. On the other hand, TGF-β pathway mutations are not common in breast and skin cancers [93]. 

Considering the dual role of TGF-β pathway in cancer, an ideal therapy would simultaneously suppress TGF-β’s tumor-promoting activity and reactivate the tumor-suppressing function of the pathway [92]. There are many TGF-β signaling antagonists under development because blocking TGF-β and its pathway offers therapeutic opportunities. Currently, there are pre-clinical and clinical data related to four major classes of these antagonists: receptor kinase inhibitors, TGF-β antibodies (ligand traps), peptide aptamers and antisense oligonucleotides [92].

**Figure 10:**
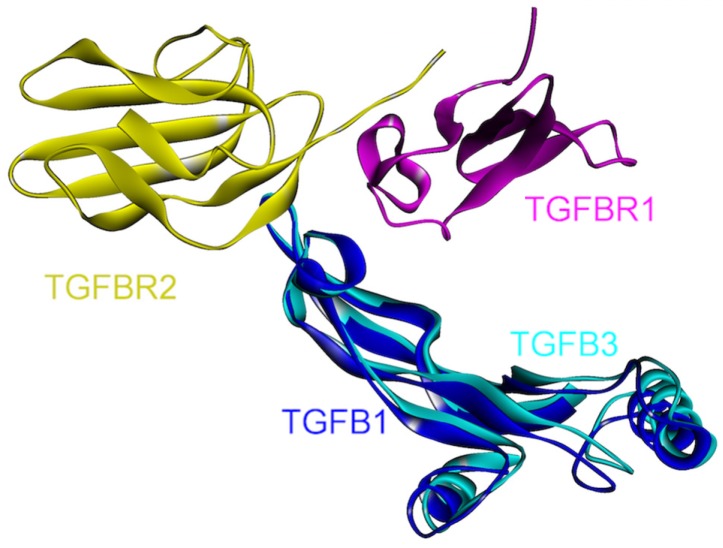
Superimposed structures of TGFβ1 and TGFβ3, in complex with TGFBR1 and TGFBR2 (PDB Code_Chains: 3kfd_BJF and 2pjy_ABC, respectively).

The 3D structures of TGF-β1-TGFBR1-TGFBR2 and TGF-β3-TGFBR1-TGFBR2 complexes are available in the PDB (PDB Code_Chains: 3kfd_BJF and 2pjy_ACB, respectively). When the structures of these complexes are aligned, it is observed that the ternary complexes are in good agreement, supporting the similarity of signaling mechanism through isoforms of TGF-β (Figure 10). There are many small molecule receptor kinase inhibitor structures in the PDB (PDB_Codes: 1py5, 1rw8, 1vjy, 2wot, 2wou, 2x7o, 3faa, 3gxl, 3hmm, 3kcf and 3tzm), targeting the protein kinase domain of TGFBR1 as it is involved in its interactions with the downstream proteins. Small molecule oral drugs targeting receptor kinases have been developed widely in the last few years [92].

### 4.6. IFN-γ

**Figure 11:**
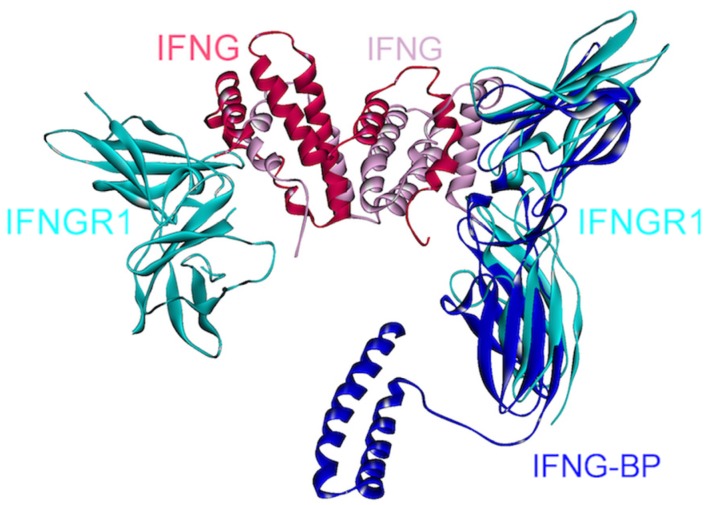
Superimposed structure of IFN-γ, IFNGR1 and viral protein IFN-γBP. A homodimer of IFN-γ interacts with two IFNGR1s (PDB Code_Chains: 1fg9_ABCD). A soluble viral protein IFN-γBP, which has a similar 3D structure with the extracellular part of IFNGR1, competes to bind to IFN-γ with IFNGR1 since they bind to overlapping interfaces on IFN-γ (superimposed structures of PDB Code_Chains: 1fg9_ABCD and 3bes_LR).

IFN-γ is a homodimeric cytokine, recognized by two separate surface-receptors, IFNGR1 (IFN-γRα) and IFNGR2 (IFN-γRβ) (Figure 1) [94,95]. Abnormal IFN-γ levels are associated with many autoimmune and auto-inflammatory diseases, liver and breast cancers [96]. In the signaling cascade, IFN-γ dimer first binds to two IFNGR1s and forms a symmetric intermediate complex (Figure 11), which subsequently recruits two chains of IFNGR2, generating an active signaling complex with a 1:2:2 stoichiometry [94]. Thus, a homodimer of IFN-γ binds to a heterotetramer of IFNGR1-IFNGR2s. Crystal structure of the complex between IFN-γ homodimer and two IFNGR1s is in the PDB (PDB_Codes: 1fg9), but the structure of the IFN-γ-IFNGR2 is currently unavailable. 

IFN-γ is an anti-viral cytokine, but some viruses, such as Ectromelia virus (ECTV) evolved a mechanism to suppress IFN-γ signaling, preventing initiation of an anti-viral host response [96]. The genome of ECTV encodes an immuno-modulatory protein, IFN-γBP (IFN-γ binding protein), which displays high structural similarity to the extracellular domain of IFNGR1. As can be seen in Figure 11, IFN-γBP competes with IFNGR1 to bind to IFN-γ, since they have overlapping interfaces on IFN-γ. This soluble viral protein mimics the receptor and disrupts its association with IFN-γ. Moreover, IFN-γBP needs to form tetramers in order to antagonize the IFN-γ pathway [96]. The reason for this requirement is currently unknown. One possibility is that monomeric interactions in the tetramer provide an optimal conformation for IFN-γ association; another may relate to higher specificity. This example demonstrates another case in which higher order oligomerization plays critical roles in signaling. Viral strategies to neutralize cytokines that are catastrophic for viral replication or action have inspired scientists to imitate viral proteins and design antagonists, which are generally composed of an antibody fused with the extracellular portion of the surface-receptor [96]. Structural details of the interactions between cytokines and such viral products serve as guidelines to achieve the aim of antagonizing the cytokines. A selective peptide inhibitor against IFN-γ and IL-10 was recently developed, employing a similar method in which the inhibitor comprises a conserved amino acid sequence of cytokine receptors [97]. In addition, inhibitors of IFN-γ have been shown to be effective in inflammatory bowel disease treatment [96,98], which is a chronic inflammatory disease and predisposes individuals to colorectal cancer [99].

## 5. Conclusions

Cytokines play important roles in cancer initiation, progression, angiogenesis, metastasis and immunotherapy. Cytokines are major players in cancer-related inflammation. Cytokines and lymphoreticular infiltrates comprise the majority of the tumor microenvironment and mediate a dialog between tumor and normal tissues. To survive, evade cancer-specific immunity and tolerate chemotherapy, tumors change the relative cytokine concentrations in the microenvironment. Different cytokine cocktails trigger different responses: while some combinations contribute to tumor initiation or promotion, others are used to activate anti-tumor immunity in cancer immunotherapy. Here, we focused on cytokines and their roles in cancer from a structural standpoint. Exploiting the crystal or modeled structures of protein-protein interactions, we illustrated that (1) some parallel pathways are mutually exclusive, like TNFR1- and TNFR2-dependent paths in TNF-α signaling, whereas others can co-exist, such as IL10 downstream paths; and that (2) oncogenic mutations that fall on the interface of protein complexes, such as E41* nonsense mutation on IL-10RB, may abolish the interaction and block the subsequent downstream signaling. We further showed that (3) oligomerization modes of proteins can affect function; and that (4) mimicking the natural antagonists of cytokines such as 2L in TNF-α signaling and viral protein IFN-γBP in IFN-γ signaling, can be an efficient method to develop novel therapeutic inhibitors of cytokines. Collectively, all aspects of cytokine signaling including their upstream and downstream pathways and crosstalk with other pathways, their interactions with receptors, oligomerization modes, sequence variants, mutations, all have a role in malignant transformation. Atomic details of cytokine pathways and interactions can help to better understand the mechanisms of how and why cytokines induce carcinogenesis. We conclude by emphasizing that structures of interactions are essential for a complete in-depth picture of cancer. 
